# The science of stress relief

**DOI:** 10.1016/j.ebiom.2022.104018

**Published:** 2022-04-21

In 2016, WHO classified stress as a modern health epidemic. 6 years later, world events have done little to alleviate stress levels. A report by WHO published in March, 2022, estimated that the COVID-19 pandemic led to a 25% increase in the global prevalence of anxiety and depression, which was largely attributable to the stress caused by extended social isolation. Stress awareness month, marked in April, affords an important opportunity to reflect on the health effects of chronic stress, as well as the scientific evidence behind popular stress-lowering interventions and coping strategies.

Stress is a universal experience. In low amounts or short durations, stress can be helpful and could serve as a motivator or to increase our resilience. Studies have shown the health benefits of short-term exposure to acute physiological stressors. According to a study in *BMC Medicine,* a short duration of thermal stress, as might be achieved through bathing in a sauna multiple times per week, was associated with significantly lower incidences of cardiovascular mortality in long-term follow up of prospective cohorts. Moreover, short-term thermal stress was also associated with a substantially reduced risk of ischaemic stroke, as reported in a 2018 study in *Neurology.* This beneficial effect was possibly driven by a reduction in inflammation and oxidative stress, as reported in a longitudinal study of 2269 men published in *Annals of Medicine*. A global survey reported in *Complementary Therapies in Medicine* found that those who used saunas more than 5 times per month also described improved mental wellbeing scores.

However, at excess levels, stress negatively affects physical and mental wellbeing. Persistent exposure to a stressor, whether psychological, physiological, or environmental, can lead to chronic inflammation and this, in turn, has a detrimental effect on health. These effects on health can include physical symptoms, such as headaches or irregular menstrual cycles, increased susceptibility to infections, and longer-term conditions, such as cardiovascular or gastrointestinal disease.

The brain in particular is vulnerable to chronic stress. Imbalance of a critical stress response pathway, the hypothalamic–pituitary–adrenal axis, results from prolonged dysregulation by stress-induced hormones, including cortisol and glucocorticoids. This process can lead to the accumulation of amyloid β-peptides and tau proteins in the brain. These proteins would usually be cleared during sleep by the activity of the glymphatic pathway and so stress-associated sleep disruptions can further compound the negative health effects of stress. Stress is a major risk factor for mental illness and increases susceptibility for neuropsychiatric conditions, such as depression and anxiety. The stress caused by mitigation measures that were introduced as a result of the COVID-19 pandemic triggered detectable levels of neuroinflammation. According to a study published in *Brain, Behavior, and Immunity* in February, 2022, changes in neuroinflammatory markers (TSPO and myoinositol) and increased levels of serum inflammatory markers (IL-16 and MCP-1), relative to measurements taken from the same individuals before the pandemic, were detected in individuals who were negative for previous SARS-CoV-2 infection. These measures correlated with higher self-reported symptom burden, such as mood alterations or mental fatigue.

Despite the negative consequences of pandemic measures on health, some have described improved wellbeing during lockdowns. In a survey of 16 940 children and adolescents aged 8–18 years published in *European Child & Adolescent Psychiatry* in February, 2022, a third of participants reported improved mental wellbeing during the first UK lockdown in 2020. Those who reported improvements in wellbeing had similar sociodemographic characteristics as the group that reported no change. Although the reasons for changes in an individual's wellbeing are multifactorial, a higher percentage of those who felt an improvement also reported having more time for exercise, getting more sleep, and positive changes in their relationships with friends and family. These observations point to the favourable effect that behavioural modifications can have on stress levels.

During lockdowns, many who were able to turned to green pursuits, such as gardening. In an international survey published in *Urban Forestry & Urban Greening* in February, 2022, 85% of respondents cited relaxation and stress-relief as an extremely important reason for them to garden. This study highlights the importance of societal provision of green spaces for wellbeing. Inflammatory biomarkers have been reported to be mediated through access to nature and residential green spaces. In a 2022 study published in *Environment International,* a prospective sampling of inflammatory markers from the blood of 7930 men and 16183 women indicated that higher neighbourhood greenness was associated with lower inflammation, independent of behavioural risk factors. A 2018 report in *Behavioural Sciences* compared biological markers of stress (cortisol and amylase) before and after visits to a wilderness-like site, municipal park, or indoor exercise facility, and found that time spent in natural environments was more beneficial in reducing physical and psychological markers of stress than exercise indoors. Similarly, a study in *JMIR Cardio* conducted in December, 2021, found that the health benefits of green spaces could be observed via metabolic and inflammatory markers associated with cardiorespiratory health, while a large prospective cohort studied published in *Environment International* in February, 2022, reported a lower incidence of inflammatory bowel disease in those with access to residential green and blue spaces. Even experiencing simulated walks through nature via virtual reality headsets can result in a significant reduction in reported stress levels, according to a pilot study of 102 health-care workers published in *PLOS One* in February, 2022.

Increasing evidence supports the benefits of mindfulness-based practices. A randomised trial published in *Brain, Behavior, & Immunity - Health* reported on an 8-week mindfulness-based programme designed to reduce stress and improve emotional self-regulation. 88 public school teachers in São Paulo (Brazil) were enrolled in the trial, with those participating in the bespoke activities reporting significantly reduced scores of perceived stress and improved psychological wellbeing compared with the control group. Reduction in serum levels of IL-6 and IL-8 and increase in levels of IL-10 and IL-12p70 were recorded after the intervention. A cross-sectional study of 17 long-term practitioners of meditation, reported in *Scientific Reports* in March, 2020, found that DNA methylation levels at specific subtelomeric regions were associated with telomere length, indicating that the long-term health benefits of meditation might have an underlying epigenetic mechanism. However, a 2021 study published in *Frontiers in Psychiatry* found that, although mindfulness-based cognitive therapy was effective in depression and anxiety-related symptom reduction in older adults, these benefits were not associated with detectable expression changes in CRP, IL-1β, or MCP-1, and gene expression levels did not differ between treatment groups.

There are many reported benefits associated with oft-practiced stress-lowering interventions, but the changes at a physiological level and underlying biological mechanisms are not fully understood. At *eBioMedicine*, we welcome robust translational studies to decipher stress response mechanisms and support the development of treatments that can alleviate the burden of stress-associated health conditions. eBioMedicine

